# Exposure to Ebola Virus and Risk for Infection with Malaria Parasites, Rural Gabon 

**DOI:** 10.3201/eid2602.181120

**Published:** 2020-02

**Authors:** Jessica L. Abbate, Pierre Becquart, Eric Leroy, Vanessa O. Ezenwa, Benjamin Roche

**Affiliations:** Institut de Recherche pour le Développement, Unité Mixte de Recherche MIVEGEC, Montpellier, France (J.L. Abbate, P. Becquart, E. Leroy, B. Roche);; Institut de Recherche pour le Développement, Unité Mixte Internationale UMMISCO, Bondy, France (J.L. Abbate, B. Roche);; CIRMF, Franceville, Gabon (E. Leroy);; University of Georgia, Athens, Georgia, USA (V.O. Ezenwa);; Universidad National Autonoma de Mexico, Mexico City, Mexico (B. Roche)

**Keywords:** Ebola virus, malaria, Plasmodium, risk factors, populations at risk, disease, ecology, epidemiology, Gabon, surveillance, prevalence, occurrence, pathogen-pathogen interactions, parasites, viruses

## Abstract

An association between malaria and risk for death among patients with Ebola virus disease has suggested within-host interactions between *Plasmodium falciparum* parasites and Ebola virus. To determine whether such an interaction might also influence the probability of acquiring either infection, we used a large snapshot surveillance study from rural Gabon to test if past exposure to Ebola virus is associated with current infection with *Plasmodium* spp. during nonepidemic conditions. We found a strong positive association, on population and individual levels, between seropositivity for antibodies against Ebola virus and the presence of *Plasmodium* parasites in the blood. According to a multiple regression model accounting for other key variables, antibodies against Ebola virus emerged as the strongest individual-level risk factor for acquiring malaria. Our results suggest that within-host interactions between malaria parasites and Ebola virus may underlie epidemiologic associations.

Major outbreaks of infections with Ebola virus, such as the 2014–2016 West Africa epidemic and the ongoing 2018–2019 outbreak in eastern Democratic Republic of the Congo, pose several obvious and immediate threats to public health. Less obvious, but as concerning for public health, is the possibility that Ebola virus might also interact with common cocirculating infectious agents at both the population and within-host (individual) levels. Indeed, much attention has been paid to the relationship between malaria and Ebola virus disease (EVD), primarily because of the clinical resemblance between the 2 diseases (*1*) and the high frequency of *Plasmodium* spp. co-infection among patients undergoing treatment for confirmed EVD (*2*). At the individual level, several retrospective epidemiology studies of patients undergoing treatment for confirmed EVD have attempted to determine whether concurrent malaria affects patient outcomes. In Sierra Leone (*3*) and at 1 Ebola treatment center in Liberia (*4*), mortality risk was much higher among Ebola patients who were co-infected with *Plasmodium* parasites than among patients who were not co-infected, and a study in Guinea found that adverse outcomes were higher among EVD patients with higher *P. falciparum* parasite loads than among those with lower levels of parasitemia (*5*). A similar study of patients at several Ebola treatment centers in Liberia reported the opposite relationship, that the probability of survival for EVD patients was positively associated with both presence and level of *Plasmodium* spp. parasitemia (*6*). Together, these results point to a strong potential for biological interactions between *Plasmodium* parasites and Ebola virus that may influence the severity of EVD.

At the population level, interruption of normal public health services and disease control measures—including patient avoidance of healthcare facilities—during an EVD epidemic has been projected to cause increases in untreated cases and deaths from malaria, in addition to several otherwise preventable or treatable diseases (*7*–*9*). Yet whether biological interactions at the within-host level, such as inflammatory processes leading to prolonged post-Ebola syndrome symptoms common in acute EVD survivors (*10*), may also lead to a change in malaria transmission dynamics by influencing susceptibility remains unknown.

Knowledge of the extent of possible interactions between infection with *Plasmodium* parasites and Ebola virus is especially helpful because geographic regions where prevalence of antibodies against Ebola virus (hereafter called Ebola antibodies) is high are also areas of high malaria endemicity (*11*), particularly the most severe form of malaria, caused by *P. falciparum* (*12*). Historically, small, typically rural, outbreaks of Ebola virus have been the norm; many such outbreaks across central Africa have been described since 1976 (*13*). However, the recent occurrence of large outbreaks involving multiple urban centers (*14*,*15*), including thousands of survivors and vaccinated persons, means that any interactions with malaria parasites have the potential to affect larger populations than in prior decades. Furthermore, it is estimated that less than half of the cross-species transmission events leading to a human EVD case are correctly identified by current surveillance systems, suggesting that most of these events are treated locally as an unknown fever or malaria (*16*).

To investigate the potential epidemiologic links between Ebola virus exposure and malaria parasites, we took advantage of a large snapshot surveillance study of 4,272 adults from 210 villages across Gabon, conducted during 2005–2008 (*17*–*19*), to test for populationwide and individual associations between the 2 infections during nonepidemic conditions. At both levels, we also tested for key cofactors that might influence detection of an association. With an Ebola antibody seroprevalence of 15.3% (*17*,*18*) and *Plasmodium* spp. prevalence of 52.1% (*19*), our study population offered the unique opportunity for testing such a link.

## Materials and Methods

### Study Population and Survey Methods

Our study was based on data previously generated from a snapshot surveillance study in rural Gabon (*17**–**21*). That study was conducted across 210 rural (population <300) villages in Gabon, located across a variety of open and forested habitats, and was designed specifically to test for the prevalence of undetected exposure to Ebola virus (*17*,*18*). Villages were selected by using a stratified random sampling method based on Gabon’s 9 administrative provinces; each province was surveyed once during 1-month field missions from July 2005 through May 2008, generally during the dry season. All but 5 of Gabon’s 49 administrative departments (grouping villages within provinces) were represented (Appendix Figure 1). In each village, all permanent residents >15 years of age were solicited for participation in the study if they were willing to complete a 2-page survey and provide a blood sample along with written consent. The survey included questions about sociodemographics and medical history. All participants and nonparticipants in each village were offered information about the study, free medical examinations, malaria testing, blood typing, and medicines. Refusal to participate was low (≈15% of eligible persons). The study protocol was approved by the Gabonese Ministry of Health (research organization no. 00093/MSP/SG/SGAQM) and is described elsewhere (*17*–*20*).

### Individual Pathogen Exposure and Cofactors

Study volunteers were tested for previous exposure to Ebola virus by use of a *Zaire ebolavirus* (ZEBOV) IgG–specific ELISA (*17*,*18*). Current infection with *Plasmodium* spp. was tested by using an in-field blood smear (*17*,*18*) and by high-throughput targeted sequencing of *Plasmodium*-specific cytochrome b mitochondrial DNA to identify species (single and mixed infections of *P. falciparum*, *P. malariae*, and *P. ovale* were identified) (*19*). For purposes of this study, we considered a person to be infected with malaria parasites if either blood smear or sequence amplification was positive (irrespective of the species) and to not be infected if both test results were negative.

In addition to participant sex and age group (16–30, 31–45, 46–60, >60 years), information was obtained about several cofactors that could be indicative of heterogeneous exposure or susceptibility to both infections (*17*,*18*). These cofactors included the presence of concurrent filarial worm infection (*Loa loa* and *Mansonella perstans*, each identified from blood samples as described in [*20*]), sickle cell hemoglobin genotype (carriers vs. noncarriers, as determined in [*21*]), participant education level (classified as less than secondary education or secondary education and above, serving as a proxy for socioeconomic status), participant regular contact with wild animals through primary occupation (classified as hunters or nonhunters), the keeping of wild animals as pets (yes or no), and specific exposure to bats by consumption (yes or no).

### Population Cofactors

For determination of population-level influences on patterns of pathogen exposure, factors common to all persons in a given department or village were also examined. We obtained population density (no. persons/km^2^) at the department level by dividing population size (no. inhabitants/department based on 2003 national census data, https://www.citypopulation.de/php/gabon-admin.php) by department area (km^2^). Average household wealth and frequency of insecticide-treated mosquito net (ITN) ownership per department were obtained from the Demographic and Health Surveys program 2012 survey for Gabon (*22*). Geographic displacement of households in these data remained within administrative boundaries; however, wealth and ITN data were missing for 7 departments (Appendix Table 1). Average household wealth was calculated by rescaling the wealth index for all rural households to positive integers and taking the geometric mean for each department. We calculated the frequency of ITN ownership per department by counting the number of rural households in each department with at least 1 ITN and dividing it by the number of households for which there were data. At the village level, the dominant habitat type was previously classified into 3 categories with statistically significant differences in terms of Ebola antibody prevalence: lakeland (including lakes, rivers and coastal regions), savanna (including savanna and grassland areas), and forest (including northeastern forests, interior forests, and mountain forest areas) (*17*,*18*).

### Statistical Analyses

We performed all statistical analyses in the R version 3.2.2 statistical programming environment (*23*). We tested for departure of malaria and Ebola antibody co-occurrence frequency from random expectations by using χ^2^ analysis (chisq.test function in R). We tested the correlation between department-level prevalence of Ebola antibodies and malaria parasite infection by using the cor.test function in R, based on the nonparametric Spearman rank correlation coefficient. We tested department-level effects of population density, average wealth, and ITN ownership frequency on this correlation together as cofactors in a mixed-effects multiple linear regression model (function lmer, package lme4) by setting Ebola antibody prevalence as the main explanatory variable, *Plasmodium* spp. prevalence as the response variable, and province as a random variable to limit pseudoreplication. The inclusion of province as a random variable also enabled us to account for yearly and seasonal differences in prevalence because all departments within a given province were sampled within a single month-long field mission. To meet assumptions of normality, antibody prevalence, *Plasmodium* parasite prevalence, and ITN ownership frequency were arcsine square-root transformed, population density and average wealth were log-transformed, and data points were weighted by the number of persons tested in each department. Data for the 7 departments with missing wealth and ITN data were excluded from the multiple regression model.

At the individual level, we used multiple logistic regression (implemented as a generalized linear mixed effects model with binomial error distribution via the *glmer* function of package lme4) to test whether persons with Ebola antibodies were more or less likely than those without Ebola antibodies to also be infected with malaria parasites. *Plasmodium* parasite infection status (infected or not infected) was the response variable in the model, and we included province (also accounting for date sampled), department within province, and village (nested within department and province) of the person as random factors to control for pseudoreplication and spatial autocorrelation. Explanatory variables included ZEBOV-specific IgG seropositivity, individual cofactors (concurrent *L. loa* and *M. perstans* infection; sex; age group; sickle cell genotype; education level; and regular interaction with animals through hunting, keeping wild pets, or consuming bats), and population-level cofactors (village habitat and log-transformed population density of the administrative department). We tested the effect of each explanatory variable after correcting for all other model terms via likelihood ratio tests, reported as adjusted odds ratios, and used bootstrapping to calculate the 95% CIs of the coefficients by using the bootMer function (R boot package, no. Markov chain Monte Carlo simulations = 200). We removed from analysis those persons for whom values for any 1 variable were missing.

## Results

**Figure 1:**
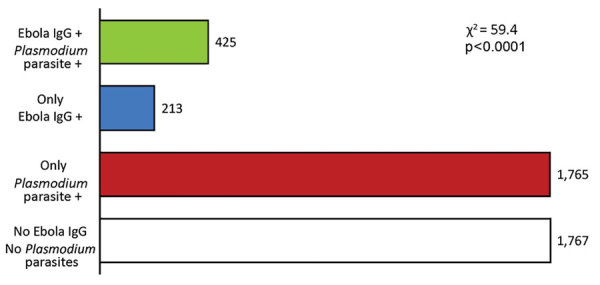
Frequency of *Plasmodium* spp. infection and *Zaire ebolavirus*–specific IgG seropositivity among participants in study of exposure to Ebola virus and risk for malaria, rural Gabon. +, positive.

A total of 4,272 volunteers from 210 villages were enrolled in the study. Among those sampled, we obtained data on both malaria status and Ebola antibodies from 4,170 persons: 2,199 (52.7%) female and 1,971 (47.8%) male participants, 16–90 (median 49) years of age. These data showed that across Gabon, 2,190 (52.5%) persons were infected with >1 species of *Plasmodium*, 638 (15.3%) were positive for ZEBOV-specific IgG, and an overabundance of 425 (10.2%) were in both categories (Figure 1; χ2 = 59.4, df = 1, p<0.0001). Because of missing data, we analyzed individual-level risk factors for exposure to both pathogens on a subset of 3,912 persons (Table; Appendix Table 1).

**Table:** Characteristics of population in study of exposure to Ebola virus and risk for malaria parasite infection, rural Gabon*

Variable	No. (%) sampled	No. with *Plasmodium* spp. infection	No. ZEBOV-specific IgG+	No. with *Plasmodium* spp. infection and ZEBOV-specific IgG+
Sex				
F	2,058 (52.6)	1,017	277	180
M	1,854 (47.4)	1,022	323	218
Age, y				
16–30	604 (15.4)	343	93	71
31–45	1,062 (27.1)	584	170	117
46–60	1,554 (39.7)	801	234	152
>60	692 (17.7)	311	103	58
Sickle cell genotype				
Carrier	811 (20.7)	424	118	83
Not carrier	3,101 (79.3)	1,615	482	315
*Loa loa*				
Infected	863 (22.1)	450	142	92
Not infected	3,049 (77.9)	1,589	458	306
*Mansonella perstans*				
Infected	391 (10.0)	230	70	48
Not infected	3,521 (90.0)	1,809	530	350
Education				
Less than secondary	2,909 (74.4)	1,478	446	292
More than secondary	1,003 (25.6)	561	154	106
Occupation				
Hunter	425 (10.9)	241	89	59
Not hunter	3,487 (89.1)	1,798	511	339
Pets				
Wild animal	450 (11.5)	263	62	46
No wild animals	3,462 (88.5)	1,776	538	352
Bat meat consumption				
Yes	522 (13.3)	273	92	53
No	3,390 (86.7)	1,766	508	345
Habitat (village)				
Forest	3,088 (78.9)	1,727	544	360
Lakeland	412 (10.5)	97	12	6
Savanna	412 (10.5)	215	44	32
Population density, department level, persons/km^2^				
0.5–2	1,936 (49.5)	995	324	210
2–10	1,379 (35.3)	702	186	124
10–30	597 (15.3)	342	90	64
*ZEBOV, *Zaire ebolavirus*; +, positive.				

**Figure 2:**
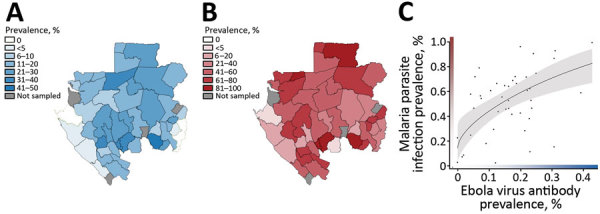
Association of Ebola virus exposure and *Plasmodium* spp. infection across rural communities in Gabon. A) Geographic distribution of Ebola virus antibody seroprevalence. B) Geographic distribution of malaria parasite (all *Plasmodium* species) prevalence. C) Correlation between these geographic distributions at the level of administrative department (ρ = 0.43, p<0.01). The fitted curve and 95% CIs (gray shading) were generated by using the predict function from the basic stats package in the R version 3.2.2 statistical programming environment (*23*), based on a linear model between the 2 variables weighted by the number of persons sampled in each department.

**Figure 3:**
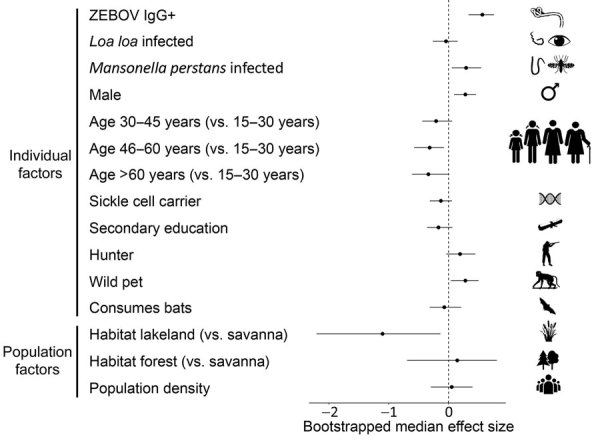
Malaria parasite infection risk factor effect sizes. The relationship between malaria and each individual or population-level risk factor was evaluated after accounting for all other variables, including geographic location (village within department within province) as a random factor, using a generalized linear mixed effects model. Effect sizes are presented as median adjusted odds ratios with bootstrapped 95% CIs. ZEBOV, *Zaire ebolavirus*; +, positive.

At the population level, we found a striking positive correlation between the geographic distributions of Ebola virus exposure and *Plasmodium* parasite infection, measured as the prevalence of each across administrative departments (Figure 2; Spearman rank correlation coefficient ρ = 0.43, df = 42, p<0.01). The direction and significance of this correlation was not qualitatively affected by population density, average household wealth, ITN ownership frequency, or by controlling for random variance among provinces sampled on different dates (Appendix Table 2, Figures 2, 3).

At the individual level, we found that prior exposure to Ebola virus was strongly associated with an increased probability of current *Plasmodium* spp. infection, even after accounting for geographic location (administrative province, department, and village) and all other individual and population-level risk factors in the model (adjusted odds ratio [aOR] 1.741 [95% CI 1.400–2.143], χ^2^ = 26.36, df = 1, p<0.0001; Figure 3; Appendix Table 3, Figure 4). This variable was a stronger risk factor for *Plasmodium* infection than any other individual trait, second only to living in a lakeland habitat (aOR 0.313 [95% CI 0.110–0.875], χ^2^ = 11.64, df = 2, p<0.01) (Figure 3; Appendix Table 3). Other factors positively associated with *Plasmodium* parasite infection were concurrent infection with *M. perstans* (aOR 1.359 [1.056–1.727], χ^2^ = 5.35, df = 1, p = 0.021), male sex (aOR 1.335 [1.098–1.586], χ^2^ = 10.5, df = 1, p = 0.0012), and keeping a wild animal as a pet (aOR 1.308 [1.040–1.654], χ^2^ = 4.55, df = 1, p = 0.033). Being in an older age group was associated with a decline in *Plasmodium* parasite infection risk (χ^2^ = 8.02, df = 1, p = 0.046). From the individual-level model we excluded department-level wealth and ITN ownership frequency, which showed no evidence for influencing the association at the population level (Appendix Table 2) because these variables were confounded with department-level population density and because missing data were not randomly distributed (Appendix Table 1). These results for nonspecific malaria parasite infection risk factors were qualitatively identical when *P. falciparum* and *P. malariae* infections were considered separately (*P. ovale* infection was too rare to be tested; Appendix Tables 4, 5).

## Discussion

At the population and individual levels across Gabon, we found a strong positive association between ZEBOV-specific IgG seropositivity and current malaria parasite infection. In geographic regions where Ebola virus exposure was high, prevalence of *Plasmodium* spp. infection was also high, and within these regions, having antibodies against Ebola virus increased the risk for current *Plasmodium* infection by nearly 75% after all other medical, demographic, social, behavioral, and ecologic cofactors for which we had data were controlled for. The magnitude of the association, particularly when compared with other risk factors (filarial worm infections, sex, age group, contact with wild animals, and village habitat type), was highly unexpected. This epidemiologic link between Ebola virus exposure and malaria is consistent with reports of high co-infection frequency during the 2014–2016 outbreak of EVD in West Africa (*2*) and suggests that ecologic processes between the 2 pathogens potentially influencing patient survival (*3*,*4*) may also influence susceptibility or transmission.

The public health implications of our findings are numerous. First, if Ebola virus infection renders patients and survivors more susceptible to malaria, healthcare providers should anticipate the need for additional malaria treatment and control measures after Ebola virus outbreaks beyond the increase predicted from disruption of healthcare services and reduced treatment-seeking behavior, which often accompany an outbreak. Second, if sublethal Ebola virus infections commonly co-occur with malaria, they may be missed because disease surveillance systems do not regularly screen for other causes of disease in *Plasmodium*-positive patients whose symptoms are consistent with malaria and resolve with malaria treatment. However, a trial in Liberia showed that antimalarial drugs inhibit Ebola virus infection of cells in culture (*24*–*26*) and were associated with increased survival of EVD patients (*4*). This finding suggests that if active treatment for malaria helps modulate EVD severity, it may also result in Ebola virus infection frequencies being underestimated during epidemic and nonepidemic periods. Third, if the causal direction of the interaction is such that malaria increases susceptibility to Ebola virus, achieving malaria elimination goals across West and Central Africa may help prevent future EVD outbreaks. Indeed, our choice to consider past exposure to Ebola virus as an explanatory variable for current malaria parasite infection in our analysis was arbitrary, and additional analyses confirmed that reversing the positions of the 2 pathogens in the model did not qualitatively change the observed association pattern (Appendix Table 6, Figures 5, 6). Furthermore, a biological mechanism of interaction between the 2 pathogens with the potential to cause the association found here (such as persistent inflammatory processes in EVD survivors [*10**,**27**,**28*] or damage to specific tissues targeted by both pathogens [*29*,*30*]) remains to be elucidated. We do, however, point out that the mechanism is not likely to be general or the result of immunosuppression (e.g., because of AIDS) because neither of the 2 common filarial infections included as co-factors (*L. loa* and *M. perstans*) were risk factors for infection with *Plasmodium* parasites (Figure 3) and Ebola virus exposure (Appendix Figure 5). Last, the World Health Organization has noted that the most recent EVD outbreak in the Nord Kivu Province of the Democratic Republic of the Congo coincided with a surge in malaria cases in the region (*31*). Even if the interaction is not biological and a common ecologic, epidemiologic, or even sociological factor not tested here is responsible for driving an increase in the probability of exposure to both pathogens, further study to identify that factor could prove helpful for predicting and preventing future EVD outbreaks.

One key challenge to understanding the drivers of the patterns we report in this study is determining what ZEBOV-specific IgG seropositivity means. Ebola virus–specific IgG is known to persist for at least a decade after acute disease (*32*). However, it is not entirely clear whether the surprisingly high seroprevalence of Ebola antibodies found in population studies such as ours during nonepidemic periods (*17*,*18*,*33*–*36*) are the result of undetected outbreaks, subclinical exposure to Ebola virus, or cross-reactivity with other unknown filoviruses. A recent modeling study estimated that nearly 75% of cross-species transmission events leading to a singular or small cluster of EVD cases go undetected (*16*), although widespread failure to detect acute EVD cases seems unlikely. Alternatively, evidence of subclinical antigenic stimulation has been documented, for example, by a survey of Ebola virus–specific IgG seroprevalence among domestic dogs. Frequency of Ebola virus–specific IgG was highest in dogs nearest to an outbreak epicenter in Gabon (*37*). Mild or asymptomatic Ebola virus infection is typically associated with low viral loads, limiting virus capacity for human-to-human transmission (*38*–*40*). Thus, evidence suggests that widespread seroprevalence of Ebola antibodies outside of known epidemic periods could reflect past subclinical infection contracted through exposure to natural reservoirs (such as frugivorous bats [*41**,**42*]); however, studies of humans have yielded only minimal support for this hypothesis (*40*,*43*,*44*). Whereas asymptomatic seroconversion of household contacts of acutely ill patients and high-risk exposure (direct physical contact with blood or vomit) was demonstrated to occur at high frequency (11/24 persons) during the 1996 outbreak in Gabon (*44*), studies from the Democratic Republic of the Congo in 1995 (*43*) and during the 2014–2016 outbreak in Sierra Leone (*40*) found that this phenomenon was much more rare among household contacts with lower-risk exposure histories. Although these studies concluded that undiagnosed subclinical EVD and asymptomatic Ebola virus infections were evident during an outbreak, it has not yet been shown that they occur in the absence of diagnosed cases, let alone at sufficient frequency. Arguably, the most likely source of high Ebola antibody seroprevalence in the absence of large outbreaks is antibody cross-reactivity with an unknown and relatively asymptomatic virus; however, whereas IgG is largely cross-reactive among Ebola virus species (*45*), no such low-virulence Ebola-related virus has been identified circulating in these populations. Irrespective of the processes that govern the presence of Ebola-specific antibodies, the strong and consistent associations we found between antibody status and *Plasmodium* parasite infection risk suggest a need for additional investigation regarding the effect of the source of these antibodies on malaria epidemiology and vice versa.

In addition to resolving uncertainty around the provenance of Ebola-specific antibodies in the absence of known cases, future studies should aim to ascertain more detailed information on the timing, duration, and severity of *Plasmodium* infections. In particular, it would be very informative to know whether the positive association detailed here is also found in children (our study excluded persons <16 years of age) because the prevalence of acquired immunity against many pathogens, including Ebola virus (*17*) and *Plasmodium* spp. (*46*), increases with age because of accumulating exposure opportunities. A longitudinal cohort (following infection and immunity status of each individual through time) would produce results with more reliable interpretation than the cross-sectional (single time-point snapshot) design of our present study (*47*). Ultimately, only case-controlled experimental studies, such as vaccine trials, can provide the evidence necessary to claim a causal relationship between these 2 pathogens in humans.

The 2014–2016 Ebola virus outbreak in West Africa served as a wake-up call, highlighting the possibility of Ebola virus emergence into new and heavily populated regions and spurring the advancement of vaccine development and case-reactive ring vaccination methods (*48*,*49*). However, with >17,000 EVD survivors across West Africa and an unknown number of asymptomatic seroconverted persons (*50*), it is important to clarify the mechanistic basis of our findings because this knowledge will help guide future investigations into public health implications, including the risk for acquiring malaria among EVD survivors and the potential for added benefits of both Ebola and malaria vaccination campaigns.

Appendix Supplementary data for study of exposure to Ebola virus and risk for malaria parasite infection, rural Gabon.
