# Supplementary material for: Exposure to Ebola Virus and Risk for Infection with Malaria Parasites, Rural Gabon
Source: Emerg Infect Dis. 2020 Feb;26(2):229–37. doi: 10.3201/eid2602.181120 (PMC6986822; doi:10.3201/eid2602.181120)
Supplement: Appendix — Supplementary data for study of exposure to Ebola virus and risk for malaria parasite infection, rural Gabon. [file 18-1120-Techapp-s1.pdf]

Article DOI: https://doi.org/10.3201/eid2602.181120

Exposure to Ebola Virus and Risk for Infection with Malaria Parasites, Rural Gabon

Appendix

Supplementary Data and Analyses

Population-level data
- Appendix Fig 1
- Appendix Table 1

Department-level co-factor analysis
- Appendix Table 2
- Appendix Fig 2
- Appendix Fig 3

Malaria infection risk analysis
- Appendix Table 3
- Appendix Fig 4

P. falciparum infection risk analysis
- Appendix Table 4

P. malariae infection risk analysis
- Appendix Table 5

Ebola virus exposure risk analysis
- Appendix Table 6
- Appendix Fig 5
- Appendix Fig 6

Appendix Table 1. Population-level data.

Province (Date Sampled)	Department	Number Villages Sampled	Number Persons Sampled	Population Size (2003 National Census)	Population Density (Persons per km2)	Average Household Wealth Score (DHS 2012)	Most Common Wealth Index Quantile (% households) (DHS 2012)	Frequency of Households with an ITN (DHS 2012)	Malaria Parasite Prevalence	ZEBOV-specific IgG Antibody Prevalence
Estuaire (July 2005)										
	KOMO	17	207	12690	1.28	-93740	poorest (87%)	0.72	72.5%	18.4%
	KOMO MONDAH	4	53	104302	28.58	8407	poorest (29.5%)	0.71	54.7%	7.5%
	NOYA	5	40	6268	1.58	-57261	poorest (76.9%)	0.94	65.0%	15.0%
Haut-Ogooué (April 2007)										
	DJOUE	1	18	3503	1.57	-119763	poorest (100%)	0.72	72.2%	5.6%
	DJOUORI AGNILI	1	24	4301	1.98	-81382	poorest (90%)	0.65	25.0%	4.2%
	LEBOMBI LEYOU	1	11	53921	20.74	-70923	poorest (86.7%)	0.65	18.2%	9.1%
	LEKABI LEWOLO	2	48	6417	5.77	-61302	poorest (80%)	0.68	66.7%	12.5%
	LEKOKO	1	14	3412	0.87				100.0%	42.9%
	LEKONI LEKORI	1	18	8978	6.79				61.1%	22.2%
	MPASSA	5	116	117768	24.00	-61645	poorest (51.1%)	0.78	44.0%	12.9%
	OGOOUE LETILI	1	12	4043	2.55				75.0%	25.0%
	PLATEAUX	1	22	9122	1.68				22.7%	0.0%
	SEBE BRIKOLO	4	78	12228	1.65	-88556	poorest (93.3%)	0.70	29.5%	11.5%
Moyen-Ogooué (January 2006)										
	ABANGA BIGNE	7	179	13178	1.64	-73425	poorest (80.9%)	0.62	55.3%	7.3%
	OGOOUE ET LACS	22	364	47812	5.27	-95132	poorest (81.7%)	0.72	46.7%	4.9%
Ngounie (June 2006)										
	BOUMI LOUETSI	2	39	22854	5.86	-122344	poorest (100%)	0.68	87.2%	30.8%
	DOLA	5	120	7858	2.97	-106293	poorest (100%)	0.72	55.8%	14.2%
	DOUYA ONOYE	1	26	23144	9.18				11.5%	23.1%
	LOUETSI BIBAKA	2	33	3680	0.98				0.0%	12.1%
	LOUETSI WANO	2	45	12849	14.45	-109729	poorest (89.7%)	0.73	93.3%	31.1%
	MOUGALABA	2	26	2571	1.01				15.4%	30.8%
	NDOLOU	2	48	5619	1.18	-44496	poorest (70%)	0.80	66.7%	18.8%
	OGOULOU	3	47	10071	1.24	-110666	poorest (87.7%)	0.74	55.3%	23.4%
	TSAMBA MAGOTSI	3	64	12769	1.27	-111173	poorest (95.5%)	0.53	71.9%	21.9%
Nyanga (January 2007)										
	BASSE BANIO	4	72	7186	2.13	-111682	poorest (95.6%)	0.75	55.6%	4.2%
	DOUIGNI	2	29	7374	3.21	-116101	poorest (90%)	0.74	79.3%	6.9%
	DOUTSILA	3	76	3920	1.38	-125031	poorest (100%)	0.76	60.5%	10.5%
	MONGO	6	105	3792	0.62	-116857	poorest (96.5%)	0.60	53.3%	16.2%
	MOUGOUTSI	7	108	26137	8.41	-120926	poorest (87.2%)	0.71	70.4%	17.6%
Ogooué Ivindo (June 2007)										
	IVINDO	11	182	27933	1.56	-125297	poorest (97.8%)	0.89	62.6%	23.1%
	LOPE	8	138	15205	1.27	-62753	poorest (68.3%)	0.93	43.5%	19.6%
	MVOUNG	4	93	5821	1.37	-105176	poorest (95%)	1.00	46.2%	23.7%

Province (Date Sampled)	Department	Number Villages Sampled	Number Persons Sampled	Population Size (2003 National Census)	Population Density (Persons per km2)	Average Household Wealth Score (DHS 2012)	Most Common Wealth Index Quantile (% households) (DHS 2012)	Frequency of Households with an ITN (DHS 2012)	Malaria Parasite Prevalence	ZEBOV-specific IgG Antibody Prevalence
	ZADIE	11	178	15204	1.56	-134170	poorest (99.2%)	0.78	46.6%	16.3%
Ogooué Lolo (September 2007)										
	LOLO BOUENGUIDI	9	266	26630	3.41	-124936	poorest (97.3%)	0.81	41.7%	17.3%
	MOULOUNDOU	5	140	26036	1.67	-53882	poorest (64.5%)	0.55	37.9%	22.9%
	OFFOUE ONOYE	1	14	5471	1.34	-127575	poorest (94.9%)	0.72	28.6%	21.4%
Ogooué Maritime (May 2008)										
	BENDJE	2	34	112424	15.02	-87398	poorest (77.5%)	0.59	2.9%	0.0%
	ETIMBOUE	4	103	6818	0.79	-91807	poorest (86.2%)	0.80	8.7%	1.9%
	NDOUGOU	4	68	9532	1.09	-91118	poorest (75%)	0.84	7.4%	1.5%
Woleu Nten (April 2006)										
	HAUT KOMO	1	25	6266	2.89	-92437	poorest (96.7%)	0.82	96.0%	20.0%
	HAUT NTEM	5	153	16843	1.12	-119190	poorest (95%)	0.71	90.8%	24.2%
	NTEM	15	376	44222	18.90	-90866	poorest (88.5%)	0.77	64.6%	16.2%
	OKANO	2	27	16447	1.67	28260	poorer (44.1%)	0.63	59.3%	40.7%
	WOLEU	11	331	73235	8.57	-95416	poorest (91.1%)	0.62	45.3%	15.4%

ITN = Insecticide Treated Net ; ZEBOV = Zaire ebolavirus antigen.

Appendix Table 2. Department-level predictors of *Plasmodium* spp. prevalence in rural Gabon. Effects of predictors were tested after first controlling for all other predictors in a mixed multiple linear regression model with province treated as a random factor. Data based on 37 departments for which all data were available.

Random effects	**No. depts**	**No. groups**	**Variance**	**Std. dev.**		
Province	37	9	0.023	0.15		
Fixed effects	Estimate	Std. err.	df	χ^2	p-value	
Intercept	1.09	0.58				
Ebola (ZEBOV)-specific IgG antibody prevalence	1.21	0.29	1	16.93	2.54e-04	***
Population density	0.0043	0.023	1	0.88	0.35	
ITN frequency	0.25	0.25	1	0.55	0.46	
Average wealth	-0.087	0.048	1	0.60	0.44	

** p<0.01 *** p<0.001.

Appendix Table 3. Contribution of past Ebola virus exposure, individual, and population factors on current malaria parasite infection including all species of *Plasmodium* (GLMM results)

**Random effects**	**No. individuals**	**No. groups**	**Variance**	**Std Dev**		
**Village**		**210**	**0.73**	**0.85**		
**Department**		**44**	**0.93**	**0.96**		
**Province**	**3912**	**9**	**0.08**	**0.28**		
**Fixed effects**	**Adjusted Odds Ratio**	**Lower CI**	**Upper CI**	χ^2	**p-value**	p
Intercept	1.042	0.452	2.238		0.918	
Ebola (ZEBOV)-specific IgG antibodies (present vs. absent)	1.741	1.400	2.143	26.36	3.83e-07	****
Loa loa infection (present vs. absent)	0.959	0.769	1.165	0.20	0.66	
Mansonella perstans infection (present vs. absent)	1.359	1.056	1.727	5.35	0.021	*
Sex (male vs. female)	1.335	1.098	1.586	10.50	0.0012	**
Age groups	0.814	0.645	1.065	8.02	0.046	*
(30-45 vs. 15-30)	0.723	0.563	0.927			
(45-60 vs. 15-30)	0.715	0.544	1.009			
(Over 60 vs. 15-30)						
Sickle cell trait (HbS) (present AS vs. absent AA)	0.884	0.733	1.059	1.70	0.19	
Secondary education (yes vs. no)	0.851	0.698	1.069	2.63	0.10	
Occupation (hunter vs. non-hunter)	1.220	0.963	1.567	2.04	0.15	
Wild animal as pet (yes vs. no)	1.308	1.040	1.654	4.55	0.033	*
Consumes bat meat (yes vs. no)	0.934	0.730	1.236	0.33	0.57	
Village habitat	0.313	0.110	0.875	11.64	2.97e-03	**
(lakeland vs. savanna)	1.160	0.500	2.245			
(forest vs. savanna)						
Population density (by department, continuous)	1.070	0.744	1.496	0.14	0.71	

* p<0.05 ** p<0.01 *** p<0.001 **** p<0.0001.

Appendix Table 4. Contribution of past Ebola virus exposure, individual, and population factors on current *P. falciparum* infection (GLMM results)

Random effects	**No. individuals**	**No. groups**	**Variance**	**Std Dev**
Village		**210**	**0.86**	**0.93**
Department		**44**	**0.76**	**0.87**
Province	**3343**	**9**	**0.18**	**0.42**

**Fixed effects**	**Adjusted Odds Ratio**	**Lower CI**	**Upper CI**	χ^2	**p-value**	**p**
Intercept	0.578	0.233	1.479		0.205	
Ebola (ZEBOV)-specific IgG antibodies (present vs. absent)	1.536	1.214	1.928	13.28	2.68e-04	***
Loa loa infection (present vs. absent)	1.028	0.843	1.263	0.069	0.79	
Mansonella perstans infection (present vs. absent)	1.423	1.046	1.924	5.95	0.014	*
Sex (male vs. female)	1.198	0.98	1.448	3.26	0.071	
Age groups	0.829	0.638	1.072	11.59	8.93e-03	**
(30-45 vs. 15-30)	0.65	0.519	0.845			
(45-60 vs. 15-30)	0.746	0.554	1.11			
(Over 60 vs. 15-30)						
Sickle cell trait (HbS) (present AS vs. absent AA)	0.841	0.695	1.045	2.71	0.09	
Secondary education (yes vs. no)	0.968	0.775	1.237	0.090	0.76	
Occupation (hunter vs. non-hunter)	1.417	1.055	1.973	5.09	0.024	*
Wild animal as pet (yes vs. no)	1.144	0.869	1.557	0.94	0.33	
Consumes bats (yes vs. no)	0.985	0.758	1.27	0.014	0.91	
Village habitat	0.278	0.091	0.837	11.20	3.71e-03	**
(lakeland vs. savanna)	1.136	0.457	2.465			
(forest vs. savanna)						
Population density (by department, continuous)	1.09	0.773	1.486	0.25	0.62	

* p<0.05 ** p<0.01 *** p<0.001 **** p<0.0001.

Appendix Table 5. Contribution of past Ebola virus exposure, individual, and population factors on current *P. malariae* infection (GLMM results)

Random effects	**No. individuals**	**No. groups**	**Variance**	**Std Dev**		
Village		**210**	**0.68**	**0.83**		
**Department**		**44**	**0.07**	**0.27**		
**Province**	**3343**	**9**	**0.0097**	**0.098**		
**Fixed effects**	**Adjusted Odds Ratio**	**Lower CI**	**Upper CI**	χ^2	**p-value**	**p**
Intercept	0.067	0.034	0.128		2.3e-12	****
Ebola (ZEBOV)-specific IgG antibodies (present vs. absent)	1.743	1.221	2.351	13.00	3.83e-04	***
Loa loa infection (present vs. absent)	1.038	0.778	1.367	0.062	0.80	
Mansonella perstans infection (present vs. absent)	0.907	0.616	1.285	0.61	0.43	
Sex (male vs. female)	1.426	1.036	2.017	6.08	0.014	*
Age groups				5.97	0.11	
(30-45 vs. 15-30)	0.693	0.49	1.015			
(45-60 vs. 15-30)	0.769	0.527	1.154			
(Over 60 vs. 15-30)	0.604	0.342	0.933			
Sickle cell trait (HbS) (present AS vs. absent AA)	1.041	0.717	1.465	0.07	0.79	
Secondary education (yes vs. no)	0.991	0.723	1.355	0.0034	0.95	
Occupation (hunter vs. non-hunter)	0.869	0.547	1.29	0.40	0.52	
Wild animal as pet (yes vs. no)	1.083	0.738	1.476	0.17	0.68	
Consumes bat meat (yes vs. no)	1.134	0.826	1.56	0.46	0.50	
Village habitat				17.71	1.43e-04	***
(lakeland vs. savanna)	0.208	0.051	0.621			
(forest vs. savanna)	1.286	0.641	2.655			
Population density (by department, continuous)	1.06	0.858	1.358	0.29	0.59	

* p<0.05 ** p<0.01 *** p<0.001 **** p<0.0001.

Appendix Table 6. Contribution of current malaria parasite infection (including all species of *Plasmodium*), individual, and population factors on Ebola (ZEBOV)-specific IgG seropositivity (GLMM results)

Random effects	**No. individuals**	**No. groups**	**Variance**	**Std Dev**	
Village		**210**	**0.039**	**0.203.79**	
Department		**44**	**1.4e-15**	**e-08**	
Province	**3912**	**9**	**0.096**	**0.31**	
**Fixed effects**	**Adjusted Odds Ratio**	**Lower CI**	**Upper CI**	**χ^2**	**p-value**
Intercept	0.072	0.04	0.117		2.00e-16
Current *Plasmodium* spp. infection (present vs. absent)	1.786	1.521	2.28	35.34	2.77e-09
Loa loa infection (present vs. absent)	1.028	0.815	1.257	0.064	0.80
Mansonella perstans infection (present vs. absent)	1.105	0.8	1.447	0.44	0.51
Sex (male vs. female)	1.262	1.022	1.589	4.68	0.031
Age groups	1.047	0.753	1.346	0.46	0.93
(30-45 vs. 15-30)	0.971	0.736	1.328		
(45-60 vs. 15-30)	0.970	0.708	1.363		
(Over 60 vs. 15-30)					
Sickle cell trait (HbS) (present AS vs. absent AA)	0.877	0.713	1.078	1.33	0.25
Secondary education (yes vs. no)	0.919	0.724	1.165	0.51	0.48
Occupation (hunter vs. non-hunter)	1.21	0.837	1.579	1.60	0.21
Wild animal as pet (yes vs. no)	0.849	0.626	1.196	1.20	0.27
Consumes bat meat (yes vs. no)	1.087	0.795	1.394	0.38	0.54
Village habitat	0.492	0.221	0.905	22.66	1.20e-05
(lakeland vs. savanna)	1.868	1.344	2.831		
(forest vs. savanna)					
Population density (by department, continuous)	0.918	0.838	1.043	2.28	0.13

* p<0.05 ** p<0.01 *** p<0.001 **** p<0.0001.

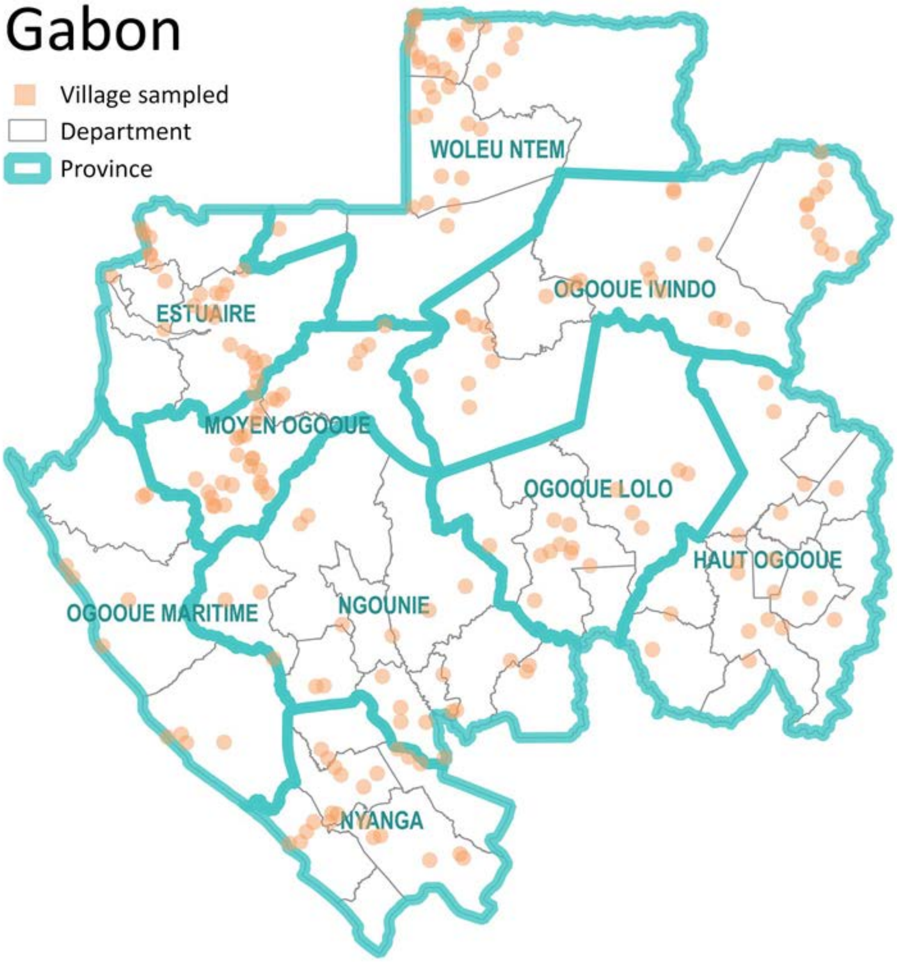

Appendix Figure 1. Map of Gabon's administrative provinces (outlined in blue, labeled by its name), departments (outlined in gray, not individually labeled), and villages (in orange) that were sampled for anti-Ebola antibodies and *Plasmodium* spp. infections.

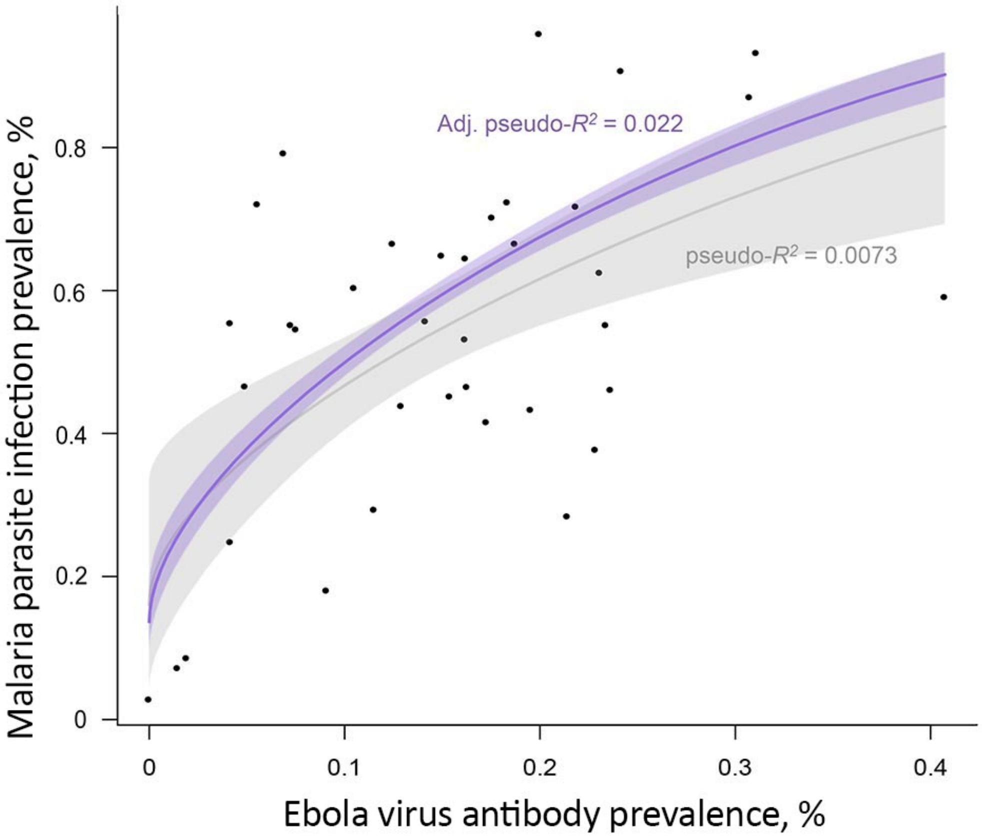

Appendix Figure 2. Relationship between prevalence of anti-Ebola (ZEBOV)-specific IgG antibodies and malaria parasite (*Plasmodium* spp.) infections in rural Gabon. The gray line and shaded area indicates the raw correlation and confidence interval (pseudo-R^2 = 0.0073), while the purple line and shaded area represent the correlation adjusted for population size, average household wealth, and frequency of insecticide treated nets per department using a mixed-effects multiple linear regression model (adjusted pseudo-R^2 = 0.022; see Appendix Table 2). Data based on 37 departments for which all data were available. Pseudo-R^2 values reflect the marginal variance explained by fixed factors in each model (using the *r.squaredGLMM* function in the 'MuMIn' package).

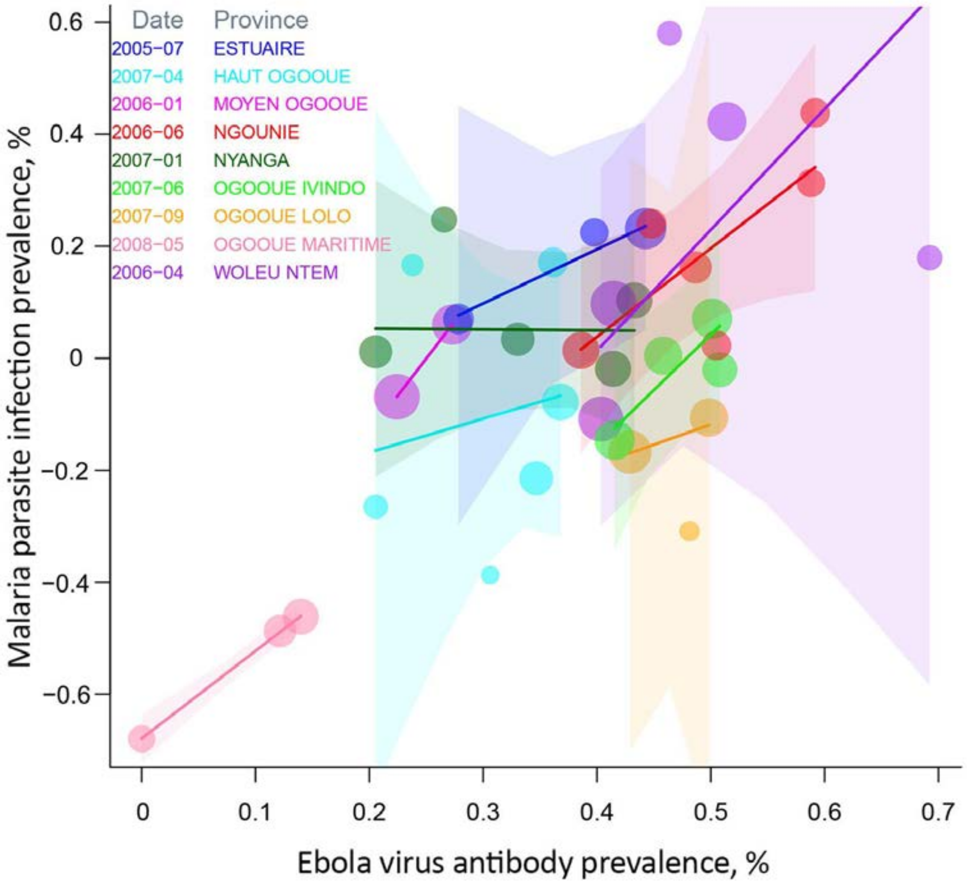

Appendix Figure 3. Relationship between prevalence of Ebola (ZEBOV)-specific IgG antibodies and malaria parasite (*Plasmodium* spp.) infections in rural Gabon by administrative province. The y-axis represents the residual variation in *Plasmodium* spp. prevalence after correcting for population density, average wealth, and frequency of insecticide-treated nets, modeled separately for departments in each province. Shaded areas represent the 95% confidence interval. Each province was sampled during a single month, given in the key as Date (year-month).

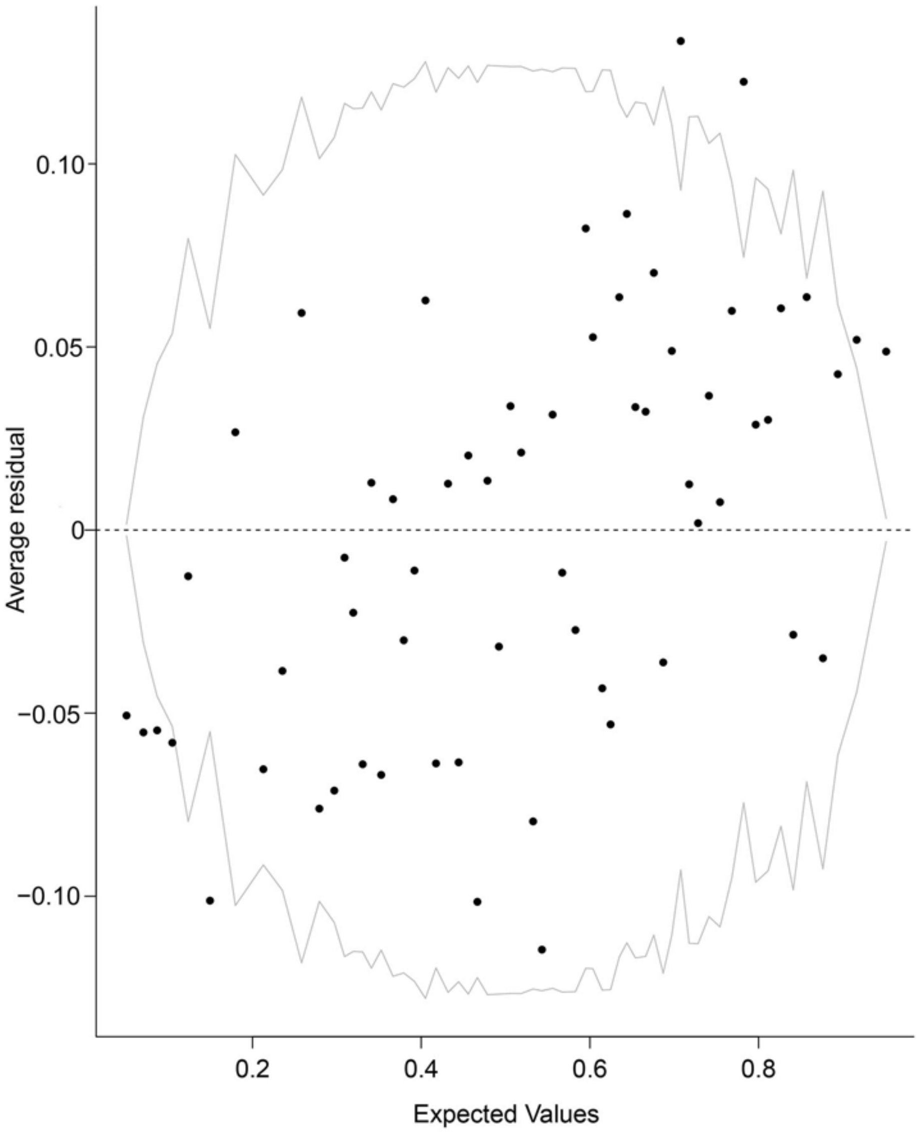

Appendix Figure 4. Model performance assessment for individual-level malaria parasite infection risk analysis (results given in Appendix Table 3, details given in the main text). Average residuals against binned expected values were plotted using the *binnedplot* function in R package ‘arm’. Gray bands represent +/- 2 standard errors. Here, 3 (5%) of 60 values truly exceed the 95% confidence window, thus not exceeding random expectations.

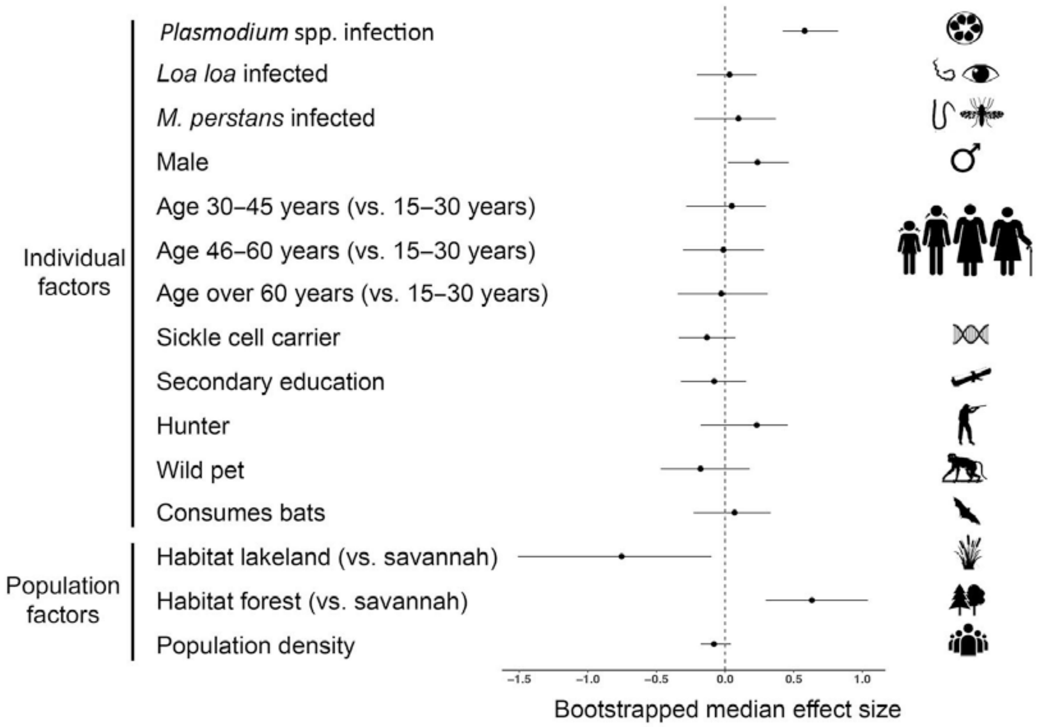

Appendix Figure 5. Ebola virus exposure risk factor effect sizes. The relationship between Ebola (ZEBOV)-specific IgG antibody presence and each individual or population-level risk factor was evaluated after accounting for all other variables, including geographic location (village within department within province) as a random factor, using a Generalized Linear Mixed Model (results in Appendix Table 6, methods in the main text). Effect sizes are presented as median adjusted odds ratios with bootstrapped 95% confidence intervals.

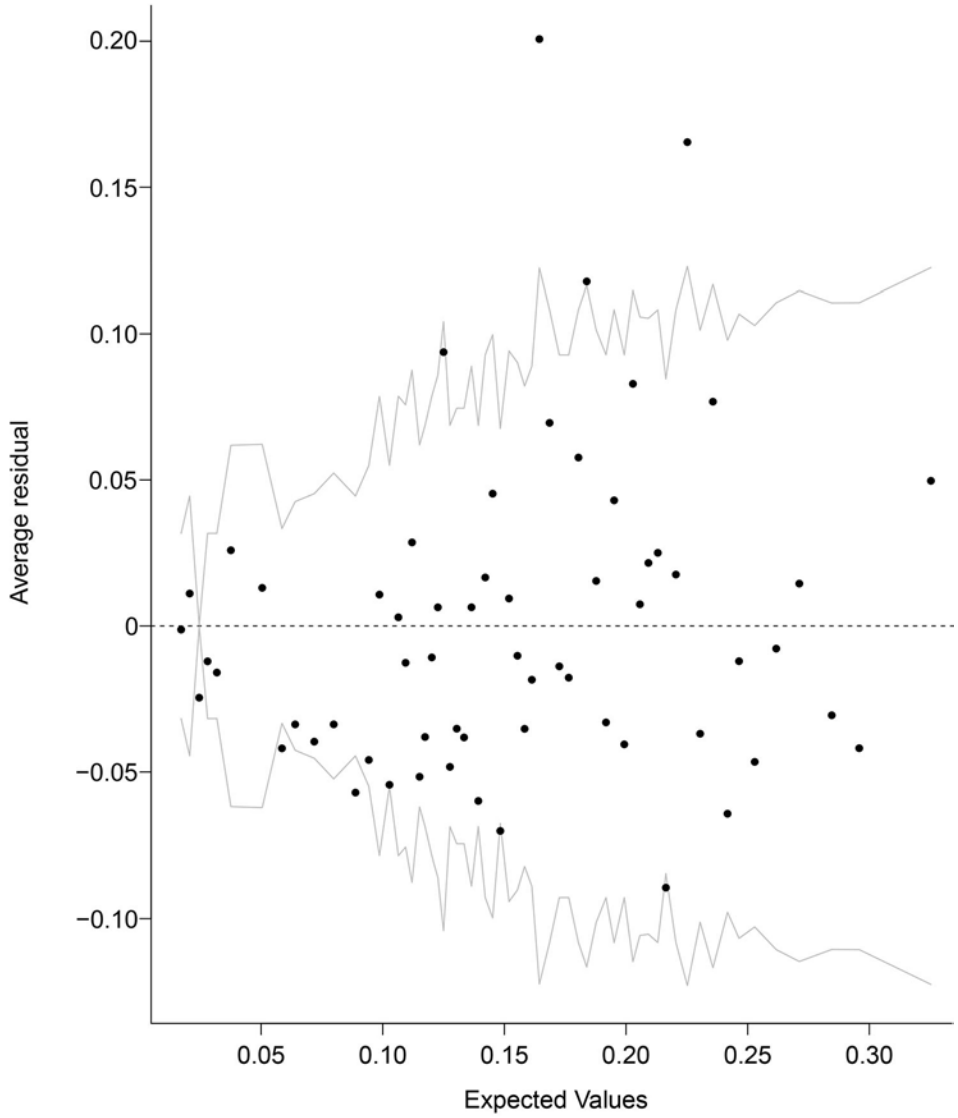

Appendix Figure 6. Model performance assessment for individual-level Ebola virus exposure risk analysis (results given in Appendix Table 6, details given in the main text). Average residuals against binned expected values were plotted using the *binnedplot* function in R package ‘arm’. Gray bands represent +/- 2 standard errors. Here, 2 (3.2%) of 62 values truly exceed the 95% confidence window, thus not exceeding random expectations.
